# Fetal sex and maternal fasting glucose affect neonatal cord blood-derived endothelial progenitor cells

**DOI:** 10.1038/s41390-022-01966-4

**Published:** 2022-02-18

**Authors:** Elisa Weiss, Barbara Leopold-Posch, Anna Schrüfer, Silvija Cvitic, Ursula Hiden

**Affiliations:** 1 Perinatal Research Laboratory, Department of Obstetrics and Gynaecology, Medical University of Graz, Graz, Austria; 2 Research Unit of Analytical Mass Spectrometry, Cell Biology and Biochemistry of Inborn Errors of Metabolism, Department of Paediatrics and Adolescent Medicine, Medical University of Graz, Graz, Austria

## Abstract

**Background:**

Maternal cardiovascular risk factors (CVRF) in pregnancy, i.e., obesity and hyperglycemia, transmit to the fetus and affect placental and fetal endothelial function. Moreover, a sex dimorphism in endothelial function and susceptibility towards CVRF exists already *in utero*. Endothelial colony-forming cells (ECFC) are circulating endothelial progenitors highly present in neonatal cord blood and sensitive to CVRF. This study investigated whether fetal sex or subtle maternal metabolic changes within healthy range alter fetal ECFC outgrowth.

**Methods:**

Outgrowth of ECFC from cord blood of male (*n* = 31) and female (*n* = 26) neonates was analyzed after healthy pregnancies and related to fetal sex and maternal metabolic parameters.

**Results:**

Male ECFC grew out earlier (−20.57% days; *p* = 0.031) than female. Although all women were non-diabetic, higher levels of fasting plasma glucose (FPG) at midpregnancy increased the time required for colony outgrowth (OR: 1.019; *p* = 0.030), which, after stratifying for fetal sex, was significant only in the males. Gestational weight gain and BMI did not affect outgrowth. Colony number was unchanged by all parameters.

**Conclusions:**

Fetal sex and maternal FPG within normal range alter ECFC function *in utero*. A role of ECFC in postnatal angiogenesis and vasculogenesis has been suggested, which may be affected by altered outgrowth dynamics.

**Impact:**

- This study is the first to report that a sexual dimorphism exists in ECFC function, as cells of female progeny require a longer period of time until colony outgrowth than ECFC of male progeny.
- Our data show that ECFC function is highly sensitive and affected by maternal glucose levels even in a normal, non-diabetic range.
- Our data raise the question of whether maternal plasma glucose in pregnancy should be considered to play a critical role even in the non-diabetic setting.

## Introduction

Endothelial cells are multifunctional cells and regulate vascular tone, angiogenesis, formation of a barrier, blood clotting, and modulate the inflammatory response.^1^ Disturbance of these functions is regarded as endothelial dysfunction (ED), which is closely associated with cardiovascular health and ED is a precursor to cardiovascular disease (CVD).^2^

Increased blood glucose, as well as overweight and adiposity, are well-established risk factors for ED and CVD.^3–7^ Hyperglycemia increases the production of reactive oxygen species and induces the formation of advanced glycation endproducts which affect endothelial function.^4,8^ Adipose tissue causes a pro-inflammatory environment through the production of cytokines, which contributes to ED.^3^ Notably, not only do pathological metabolic derangements lead to ED, even subtle changes within normal, healthy range alter the function of endothelial cells in adults. For instance, in non-diabetic, normoglycemic subjects, higher fasting plasma glucose (FPG) associates with altered endothelial function and markers for ED,^9,10^ and modest weight gain around 4 kg impairs endothelial function in women.^11^

During pregnancy, maternal cardiovascular risk factors (CVRF) transmit to the fetal compartment: Maternal obesity in pregnancy is associated with a pro-inflammatory fetal environment^12,13^ and maternal hyperglycemia directly transfers to the fetus,^14^ and both may affect the fetal and placental endothelium. In fact, gestational diabetes (GDM) alters microvascular architecture in the placenta^15^ and in the neonatal iris,^16^ and maternal GDM and obesity alter gene expression and function of endothelial cells from the placenta and umbilical cord vein.^17–21^ A recent study has revealed that not only the metabolic derangement of GDM, but also moderate metabolic alterations of maternal overweight affect placental endothelial cells and alter protease expression, suggesting that the placental and fetal endothelium may be responsive to subtle maternal metabolic changes.^13^

Endothelial function and susceptibility towards CVRF differ between the sexes.^22^ This sexual dimorphism contributes to a higher incidence of CVD reported in males^23^ and a more frequent occurrence of microvascular dysfunction in females.^24^ In adults, estrogen is regarded as the main cause underlying this difference. However, an increasing body of evidence demonstrates that a sexual dimorphism in endothelial function exists already *in utero*. In fact, in previous studies, we have revealed a sex dimorphism in miRNA expression and actin organization of feto-placental endothelial cells.^25^ Moreover, HUVEC isolated from male vs female donors reveal a sex dimorphism in endothelial nitric oxide synthase (eNOS) protein levels.^26^ In early development, genetic and epigenetic reasons are likely the main drivers of sexual dimorphism. These may involve transcripts located on sex chromosomes that encode genes associated with cardiovascular function^27^ or which may regulate the expression of autosomal genes participating in endothelial function.^28^ Notably, the fact that changes in miRNA profiles of placental endothelial cells after exposure to GDM differ in male vs female fetuses indicates that the response of endothelial cells to maternal CVRF differs depending on fetal sex.^29^

Endothelial colony-forming cells (ECFC) are circulating endothelial progenitor cells which are recruited for endothelial repair, vascular growth, and angiogenesis^30^ and give rise to cells of mature endothelial phenotype.^31^ Fetal and neonatal period is characterized by a particularly high number of circulating ECFC, probably due to massive angiogenesis and vascular remodeling.^32^ Colony outgrowth is a critical parameter reflecting ECFC function^33,34^ and requires attachment of progenitor cells to the surface, i.e., the vessel wall in vivo or the cell culture dish in vitro, differentiation into mature endothelial cells, and proliferation to form colonies. ECFC number and function are sensitive to CVRF^35–37^ and also maternal diabetes in pregnancy affects cord blood-derived ECFC.^38,39^ Whether ECFC are sensitive to metabolic characteristics within the normal, non-diabetic range is still elusive.

In this study, we addressed the question whether, within healthy, non-diabetic pregnancy, fetal sex, and maternal metabolic hallmarks, i.e., FPG and post-load glycemia at midpregnancy, gestational weight gain, and pre-pregnancy BMI, affect neonatal ECFC function. Therefore, we isolated ECFC from umbilical cord blood of male and female neonates after a healthy pregnancy and related ECFC outgrowth, i.e., the days required for colony outgrowth, the number of colonies, and the days required for confluency, to these maternal metabolic characteristics.

## Methods

### Study cohort

The present study was conducted at the Department of Obstetrics and Gynaecology at the Medical University of Graz, Austria, in accordance with the Declaration of Helsinki. Isolation of ECFC from umbilical cord blood was approved by the local ethics committee (29-319 ex 16/17) and written informed consent was obtained from all participants. A total of 57 participants were enrolled after delivery of singleton pregnancy (37–42 weeks of gestation) and cord blood samples of male (*n* = 31) and female (*n* = 26) neonates were obtained. Maternal and infant characteristics are shown in Table 1. Exclusion criteria included GDM (diagnosed by a 75 g oral glucose tolerance test (oGTT) at 24–28 weeks of gestation^40^ and defined by at least one glucose value being above the cut-off levels (92, 180, and 153 mg/dL at 0, 60, and 120 min after glucose intake, respectively)), smoking (self-reported), medical disorders or pregnancy complications (except thyroid dysfunction), use of any medication (except thyroid hormones), or adverse medical history. Two included subjects were diagnosed with polyhydramnios and in one case pregnancy was initiated by in vitro fertilization.

**Table 1:** Maternal and infant main characteristics of ECFC donors.

Characteristics	Entire cohort	Male	Female
Number of subjects	57	31	26
Maternal age (year)	31.1 ± 4.9	31.6 ± 5.4	30.5 ± 4.3
Pre-pregnancy BMI (kg/m^2^)	24.7 ± 4.3	24.4 ± 4.5	24.9 ± 4.0
BMI at delivery (kg/m^2^)	29.5 ± 4.3	29.7 ± 4.4	29.2 ± 4.1
Gestational weight gain (kg)	13.8 ± 6.0	14.5 ± 6.9	12.8 ± 4.3
oGTT 0 h (mg/dL)	80.3 ± 6.5	80.1 ± 6.7	80.5 ± 6.4
oGTT 1 h (mg/dL)	118.4 ± 28.3	110.9 ± 29.6	127.3 ± 24.4*
oGTT 2 h (mg/dL)	97.1 ± 22.8	93.1 ± 22.9	102.0 ± 22.0
Gestational age at delivery (week)	39.5 ± 1.1	39.4 ± 1.0	39.7 ± 1.2
Mode of delivery (vaginal/C-section)	22/35	10/21	12/14
Neonatal weight (g)	3513.8 ± 401.5	3535.7 ± 394.8	3487.7 ± 415.6
Neonatal height (cm)	51.2 ± 2.2	51.5 ± 2.1	50.8 ± 2.3
Placental weight (g)	649.5 ± 130.2	662.7 ± 127.7	634.2 ± 133.9

Data are presented as mean ± SD. Statistical differences are calculated by unpaired Student’s *t*-test comparing male vs female.

*oGTT* oral glucose tolerance test, *C-section* cesarean section.

*p < 0.05.

### Isolation and culture of ECFC

ECFC were isolated as described previously.^41^ In brief, 8–16 mL of venous cord blood was collected in lithium heparin-coated tubes (Greiner Bio-One, Kremsmünster, Austria) immediately after delivery of the placenta. Mononuclear cells (MNC) were separated via density gradient centrifugation using Lymphoprep™ Density Gradient Medium (Axis Shild, Alere Technologies AS, Oslo, Norway). The emerged buffy coat was washed and resuspended in culture medium (Endothelial Cell Growth Medium MV Kit (PromoCell, Heidelberg, Germany) supplemented with 0.1% Gentamycin (ThermoFisher Scientific, Waltham, MA)). 2 × 10^7^ cells were seeded per well of six-well culture plates (ThermoFisher Scientific) pre-coated with rat tail collagen type 1 (Corning, Corning, NY) and cultured at 37 °C, 21% O_2_, 5% CO_2_ in a humidified incubator. After overnight incubation, the culture medium was changed and then twice a week.

### ECFC outgrowth

**Fig. 1:**
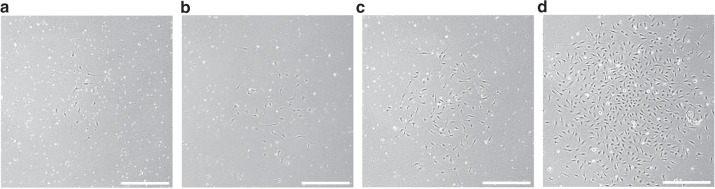
Colony outgrowth of cord blood ECFC. Microscopic photography of a representative colony using an inverted microscope from day 4 (**a**), day 5 (**b**), day 6 (**c**) until day 7 (**d**) after plating of MNC. Scale bar = 500 µm.

Colony outgrowth of ECFC was monitored daily using an inverted microscope (Olympus CKX53, Tokyo, Japan). Following the seeding of MNC, a major part of ECFC isolations formed colonies after 4–6 days. Cells revealed the typical cobblestone morphology of endothelial cells. Figure 1 illustrates the outgrowth of a representative ECFC colony followed over 4 days. The time until the first colonies formed, the number of colonies as well as the time until cells reached confluency for passaging, was recorded. For passaging, the culture medium was aspirated, cells washed with 1 × Hank’s Balanced Salt Solution (HBSS, ThermoFisher Scientific), and detached using TrypLE^TM^ (recombinant cell-dissociation enzyme, ThermoFisher Scientific). Cells were resuspended in culture medium and transferred to gelatine pre-coated (1% porcine skin gelatine (Sigma-Aldrich, St. Louis, MO) + 1% Gentamycin (ThermoFisher Scientific)) culture flasks (ThermoFisher Scientific). Colony number was related to the amount of cord blood used for isolation. Supplementary Fig. 1 represents the schematic study design.

### Characterization of ECFC via flow cytometric analysis

Phenotype of ECFC was evaluated by flow cytometric analysis (FACS) as described^41^ in 6 representative ECFC isolations. In brief, ECFC were detached, washed and stained with pre-titrated volumes of fluorochrome-conjugated antibodies (Supplementary Table 1) for 20 min at 4 °C. Measurements were performed on a CytoFLEX flow cytometer (Beckman Coulter, Brea, CA) using the CytExpert software (Beckman Coulter). For controls, unspecific antibodies of the same isotype and concentration were used. Data were analyzed using the Kaluza Analysis software (Beckman Coulter).

### Characterization of ECFC via immunocytochemistry

For quality and purity control, each ECFC isolation was subjected to immunocytochemical characterization using antibodies against endothelial cell markers (CD31, von Willebrand factor (VWF)), fibroblast markers (CD90, TE-7) and muscle cell markers (smooth muscle actin (SMA), Desmin) as described previously.^41^ In brief, cells were seeded on glass chamber slides (ThermoFisher Scientific) and fixed with acetone (Merck). TBE pH 8.0 (Gatt-Koller, Absam, Austria) with 0.1% Tween (Sigma-Aldrich) was used as rehydration and washing buffer. Primary antibodies (Supplementary Table 2) were applied for 30 min and visualization was performed with the Ultra Vision horseradish peroxidase Polymer Kit (ThermoFisher Scientific). Images were generated using a light microscope (Olympus BX53) with the UC90 camera (Olympus) and the corresponding cellSens Standard software (Olympus).

### Network formation assay on Matrigel

2D network formation ability of ECFC was analyzed in a subcohort of 23 ECFC donors (12 male, 11 female). ECFC were resuspended in culture medium supplemented with 5% FCS but without additional growth supplements, and seeded in a density of 10^4^ cells per well on 96-well culture plates (Costar, Corning), pre-coated with growth factor reduced Matrigel (Corning). Plates were incubated at 21% O_2_ at 37 °C and monitored for 24 h using an inverted phase-contrast microscope (Cell Observer; Zeiss, Oberkochen, Germany) with a digital camera and the AxioVision software V 4.8. Images at 3, 6, 12, and 24 h were quantified using the AngioJ-Matrigel assay plugin in ImageJ, V 1.43l (https://imagej.nih.gov/ij/index.html, RRID:SCR_003070) as described previously.^42^ The assay was performed in triplicates per individual donor.

### Statistics

Statistical analyses were performed using the IBM SPSS Statistics software, V25 (https://www.ibm.com/products/spss-statistics, RRID:SCR_019096). Maternal and infant characteristics, as well as FACS results, are presented as mean ± standard deviation (SD). Statistical differences in subjects’ characteristics were calculated by unpaired Student’s *t*-test. Data of Matrigel assay are presented as mean ± SD and were analyzed by unpaired Student’s *t*-test and Pearson correlation, respectively. Visual assessment with *QQ*-plots revealed that data describing ECFC outgrowth were not normally distributed. Potential confounding factors were identified via Mann–Whitney *U* test. Data are presented as median ± interquartile range (IQR). For other analyses, skewed data of the outcome/dependent variable were log-transformed and re-transformed by exponentiation for the presentation of results. Differences between groups were tested by ANCOVA (analysis of covariance), and correlation with specific parameters by linear regression analysis, in both adjusting for delivery mode. Results of ANCOVA are presented as estimated marginal means (EMM) and 95% confidence intervals (CI). Linear regression analysis revealed the unstandardized regression coefficient *B*, which requires interpretation as odds ratio (OR), i.e., percentage of increase/decrease of the dependent variable per unit increase of the independent variable, due to re-transformation of log-transformed data. Results are given by OR and 95% CI. A *p*-value < 0.05 assumed statistical significance. Graphs were generated with the GraphPad Prism software, V9 (http://www.graphpad.com, RRID:SCR_002798) using untransformed and unadjusted data, unless otherwise specified.

## Results

### ECFC phenotype

**Fig. 2:**
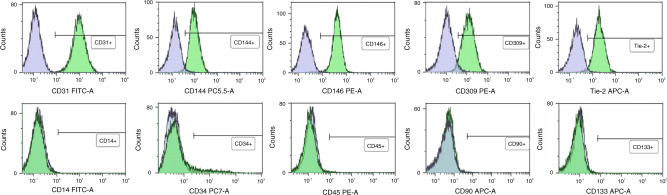
Phenotype of cord blood ECFC as determined by flow cytometry. Cells were analyzed for surface expression of the endothelial cell markers CD31 (PECAM-1), CD144 (VE-cadherin), CD146 (MCAM), CD309 (KDR), and Tie-2, and for markers for myeloid cells (CD14), hematopoietic stem cells (CD34), leukocytes (CD45), fibroblasts (CD90) and stem cells (CD133). Depicted plots represent typical expression patterns and show specific antibody staining (green peak) vs corresponding isotype control (blue peak).

ECFC phenotype was assessed by FACS analysis and immunocytochemistry. FACS analysis was performed with a subset of 6 ECFC isolations. Analysis revealed that cells were positive for the endothelial cell markers CD31 (PECAM-1), CD144 (VE-Cadherin), CD146 (MCAM), and Tie-2, and did not express markers for myeloid cells (CD14), leukocytes (CD45), fibroblasts (CD90) and stem cells (CD133). The majority of cells (90.42 ± 1.44%) were positive for the endothelial cell marker CD309 (KDR). A small population (8.38 ± 7.42%) was positive for the endothelial and hematopoietic progenitor marker CD34 (Fig. 2). Immunohistochemistry confirmed the expression of endothelial cell markers (CD31 and VWF), and the absence of fibroblast markers (CD90 and TE-7) and muscle cell markers (SMA and Desmin) in all ECFC isolations (*n* = 57) (Supplementary Fig. 2).

### Identification of confounding factors

To identify confounding factors, the influence of maternal and neonatal characteristics was analyzed. Delivery mode strongly affected the number of outgrown ECFC colonies (vaginal delivery: 5.35 ± 14.52, cesarean section (C-section): 1.31 ± 5.93, *p* = 0.001, Supplementary Fig. 3A). Although the effect of delivery mode on the number of days until initial colony outgrowth (vaginal delivery: 5.00 ± 3.00, C-section: 5.00 ± 3.00, *p* = 0.136, Supplementary Fig. 3B) did not reach significance, the days until reaching confluency was affected (vaginal delivery: 10.00 ± 2.00, C-section: 12.00 ± 3.00, *p* = 0.020, Supplementary Fig. 3C). Thus, for all further analyses, data were corrected for delivery mode.

### Effect of fetal sex and maternal metabolism on ECFC outgrowth and network formation

**Fig. 3:**
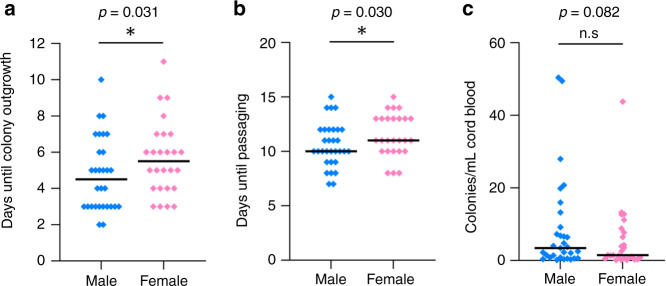
Effect of neonatal sex on ECFC colony outgrowth. **a** Days required for colony outgrowth in ECFC of male and female neonates. **b** Days required for reaching confluency in ECFC of male and female neonates. **c** ECFC colony numbers of male and female neonates. Data were analyzed via ANCOVA adjusted for delivery mode after log-transformation of the dependent variable. Graphs represent untransformed and unadjusted data with median. *n* (male) = 31, *n* (female) = 26.

First, we investigated the effect of fetal sex on ECFC outgrowth comparing isolations from 31 male vs 26 female neonates. Indeed, ECFC of female neonates required a longer period of time until colony outgrowth (male: 4.29 d (3.72; 4.93), female: 5.40 d (4.63; 6.28), *p* = 0.031, Fig. 3a) and a longer time period until reaching confluency (male: 10.30 d (9.66; 10.99), female: 11.46 d (10.69; 12.27), *p* = 0.030, Fig. 3b) than ECFC of male neonates. Similarly, the number of colonies per mL cord blood used for isolation was by trend greater in male ECFC (male: 3.30 colonies/mL (1.96; 5.56) vs. female: 1.67 colonies/mL (0.94; 2.94), *p* = 0.082, Fig. 3c).

In order to investigate whether not only outgrowth, but also the ability of ECFCs to form networks differs between the sexes, we performed a 2D Matrigel assay using a subset of 23 ECFC isolations (12 from male, 11 from female neonates). However, within this subset, there was no difference at any of the time points examined, i.e., 3, 6, 12, and 24 h, regarding the number of branching points and tube length (Supplementary Fig. 4 and Supplementary Table 3).

**Fig. 4:**
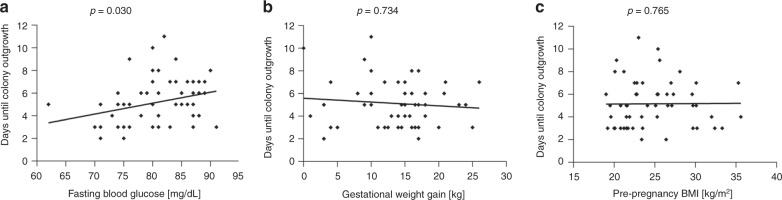
Effect of maternal metabolic parameters on ECFC colony outgrowth. **a** Positive correlation of days required for ECFC outgrowth with maternal fasting plasma glucose. **b** Absent correlation of ECFC outgrowth with maternal gestational weight gain and **c** pre-pregnancy BMI. Data were analyzed via linear regression analysis adjusted for delivery mode after log-transformation of the dependent variable. Fasting plasma glucose was determined during oGTT at gestational weeks 24–28. Graphs represent untransformed and unadjusted data. *n* = 57.

In the second analysis of outgrowth, we determined the effect of maternal metabolic parameters, i.e., FPG and glycemia after glucose challenge as well as gestational weight gain and pre-pregnancy BMI, on ECFC outgrowth. Analysis of the relation between glucose levels determined by an oGTT at midpregnancy (gestational week 24–28) and outgrowth of ECFC isolated after birth revealed a positive correlation between the number of days required for colony outgrowth with maternal FPG (OR: 1.019 (1.002; 1.035), *p* = 0.030, Fig. 4a). For instance, a 10 mg/dL increase in FPG prolongs colony outgrowth by 20%. Maternal FPG was neither associated with ECFC colony number (OR: 0.971 (0.912; 1.033), *p* = 0.353) nor with days until passaging (OR: 1.005 (0.998; 1.014), *p* = 0.189). There was no effect of plasma glucose levels after the glucose challenge on ECFC outgrowth (not shown). Moreover, neither gestational weight gain (range 0–26 kg) nor maternal pre-pregnancy BMI (range 19.0–35.6 kg/m^2^) had an effect on the days required for ECFC outgrowth (OR: 0.998 (0.977; 1.016), *p* = 0.734, Fig. 4b and OR: 0.995 (0.971; 1.023), *p* = 0.765, Fig. 4c, respectively).

Since we found an effect of FPG on ECFC outgrowth, we further analyzed whether network formation on Matrigel was also affected by maternal glycemia. However, in the subset of 23 ECFC isolations, there was no correlation of network formation with FPG (Supplementary Table 3).

**Fig. 5:**
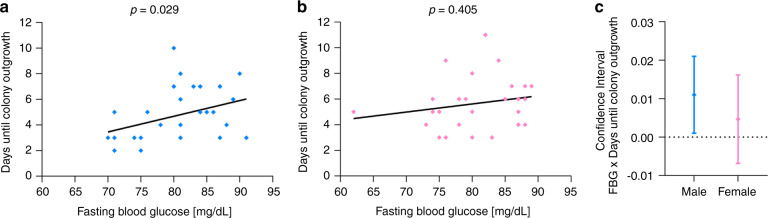
Effect of maternal FPG on ECFC colony outgrowth depends on fetal sex. **a** Positive correlation between days required for ECFC outgrowth and maternal FPG in male subjects. **b** Absent correlation between ECFC outgrowth and maternal FPG in female subjects. **c** Confidence intervals from the linear regression analysis between maternal FPG level during pregnancy and required days until neonatal ECFC colony outgrowth revealed that the correlation is only significant in the male group. Fasting plasma glucose was determined during oGTT at gestational weeks 24–28. Data were analyzed via linear regression analysis adjusted for delivery mode after log-transformation of the dependent variable. Graphs in **a** and **b** represent untransformed and unadjusted data. Graph in **c** represents log-transformed and adjusted data. *n* (male) = 31, *n* (female) = 26.

As we identified a sexual dimorphism in colony outgrowth in our first analysis, we further investigated whether the impact of FPG or any other of the investigated parameters (post-load glycemia, pre-pregnancy BMI, gestational weight gain) also depended on fetal sex. After stratifying for fetal sex, the correlation between FPG and ECFC outgrowth, i.e., days until colony outgrowth, remained significant only in ECFC of male (OR: 1.026 (1.002; 1.050), *p* = 0.029, Fig. 5a), but not in ECFC of female neonates (OR: 1.012 (0.984; 1.038), *p* = 0.405, Fig. 5b). This difference is illustrated by plotting the respective confidence intervals in Fig. 5c. The other parameters showed no dependence on neonatal sex (Supplementary Table 4).

## Discussion

We here investigated the effect of fetal sex and influence of maternal metabolic parameters within healthy pregnancy on the outgrowth of neonatal ECFC. Our key findings were that ECFC of female progeny required a longer period of time until colony outgrowth than ECFC of male progeny. Moreover, we identified that, even within the healthy, non-diabetic range, higher maternal FPG present at midpregnancy prolongs the number of days required for colony outgrowth in ECFC of male neonates.

Due to the fact that ECFC represent a tiny cell population within the mass of circulating MNC, clear quantification by FACS analysis requires a previous enrichment step, which could reduce the accuracy. Also, the use of polychromatic flow cytometry to quantify ECFC was reported^43^ although data of ECFC quantification were not shown. Therefore, the number of colonies formed in vitro is widely used as a measure for ECFC frequency. As an approximation for EPC number, Fadini et al. counted circulating CD34^+^/KDR^+^ cells in male and female individuals of different ages, including umbilical cord blood ECFC.^44^ The CD34^+^/KDR^+^ phenotype is not exclusive for EPC and may include also cells that are not of endothelial progeny and thus, do not form endothelial colonies,^45^ but results revealed that female neonates, as well as pre-menopausal women, possess higher levels of CD34^+^/KDR^+^ cells.^44^

Several studies investigated sex differences in ECFC colony formation in adults. Comparing colony outgrowth of adult ECFC in postmenopausal women and men of similar age, Hoetzer et al. reported 150% higher colony-forming capacity in women^46^ and Fadini et al. observed a higher number of ECFC colonies in female pre-menopausal adults compared to age-matched males.^44^ By contrast, Shaw et al. observed a reduced number of ECFC colonies in adult females.^47^ In parallel, also Xiao et al. obtained a lower number of EPC in females, which was determined by counting the single endothelial-like cells attached after 5 days in culture, but no sex-difference in the number of colonies formed.^48^ Interestingly, Randolph et al. identified lower differentiation capacity of female human pluripotent stem cells (hPSC) into endothelial progenitors^49^ pointing at a possible role of differentiation capacity underlying the slower outgrowth of female cells. We did not find studies investigating ECFC outgrowth during the perinatal period or in children.

We also investigated the effect of neonatal sex on in vitro network formation of ECFC. In a subcohort of 23 cases, we observed no difference between ECFC from female vs male neonates. Comparing isolated ECFC from 25 women and 25 men around the age of 60, Hoetzer et al. employed a Boyden chamber to investigate migratory activity and observed a 40% increased migration in the female group.^46^ However, we did not find any reports on the effect of sex on network formation of ECFC or of other endothelial cells.

Estrogen is a major protector against ED in adult, pre-menopausal females^50,51^, and a central role of sex hormones in the regulation of EPC is suggested.^44^ The fetal gonads start to produce steroid hormones already in early pregnancy,^52^ but a large cohort study observed no difference in intrauterine estrogen levels between boys and girls.^53^ Nonetheless, also other factors may underlie the sex differences in ECFC function. Intrinsic sex differences are based on genetic mechanisms that are directly or indirectly associated with sex chromosomal gene expression, or on epigenetic mechanisms. Such intrinsic sex differences become increasingly investigated and acknowledged.^54^ In addition, systemic sex dimorphism in growth factors, hormones, and cytokines may prime EPC and contribute to sex differences in ECFC outgrowth as a sexual dimorphism exists for several bioactive molecules present in the fetal circulation: For instance, cord blood levels of insulin-like growth factor 1 (IGF-1) and of leptin are lower in male than in female neonates.^55,56^

Besides the effect of fetal sex, we investigated the impact of maternal metabolism on neonatal ECFC. Hyperglycemia is a major cause of ED in diabetes^57^ and the effect of GDM on neonatal ECFC has been investigated in several settings: Studies observed fewer^38^ or unchanged^39,43^ ECFC colonies after GDM pregnancies with no difference in days until colony appearance,^38^ and revealed both, reduced^38^ and increased proliferation^39^ of ECFC after GDM.

In the subcohort used for Matrigel assays, we did not observe an effect of maternal glycemia on ECFC network formation. Ingram et al. has shown a strong negative effect of maternal diabetes on network formation of cord blood ECFC, although the sample size investigated was quite small with 5 controls and 4 diabetic subjects.^58^ The fact that we observed no effect of maternal glucose levels on ECFC network formation may thus be to the fact that all ECFC donors are non-diabetic and potential subtle changes are too small to be identified within a cohort of this size.

When we analyzed samples of our healthy, non-diabetic cohort according to maternal FPG at midpregnancy, we discovered that increasing glucose levels prolonged the period of time required for outgrowth. FPG represents the value to which the body can lower blood glucose before meals, and pathologically elevated FPG in pregnancy represents a much stronger predictor for adverse pregnancy outcomes than elevated post-load glucose.^59^ Our data indicate that even within the normal range, higher levels of FPG affect the fetus, which parallels a study from Liu et al., who demonstrated an association between maternal FPG at gestational weeks 10–24 with birth weight.^60^ Authors concluded that maternal fasting glycemic state affects fetal development. Of note, this correlation was more pronounced in male than in female neonates. Also in our study, the effect of maternal FPG on fetal cells was sex-dependent, since the correlation with colony outgrowth was only significant in ECFC from male neonates. Besides, Liu et al. demonstrated, that neonatal birth weight was only associated with fasting glucose level measured during oGTT, but not with glycemia after the glucose challenge, highlighting the role of maternal basal blood glucose on fetal development.^60^ Again, this result is in accordance with ours, as we also did not detect a correlation between ECFC outgrowth and glycemia after the glucose challenge. In our study, we did not identify an association between FPG and birth weight, which may be due to a much smaller sample size of our study. The study by Liu et al. investigated a cohort of 2284 cases, whereas our cohort included 57 subjects, making such correlations more difficult to detect.

Our data highlight the fact that endothelial function is subject to sex dimorphism. Whilst in adults and due to differences in sex hormones, females possess a better endothelial function and are at lower risk to develop cardiovascular disease, neonatal ECFC reveal a better outgrowth in males. This highlights the fact that intrinsic, genetic, and epigenetic sex differences differ from acquired sex differences as a result of hormones and lifestyle. Our data, however, further revealed that male neonatal ECFC are more sensitive to metabolic influences as outgrowth correlated with maternal FPG within the normal range. A higher susceptibility towards environmental risk factors, even when subtle, may contribute to a higher risk for CVD in male adults. ECFC outgrowth is sensitive towards CVRF in adults and neonates.^35–39,61^ Whether slower ECFC outgrowth is assigned of adverse effects of CVRF on any exposed endothelial cell type, or whether ECFC dysfunction in fact contributes to CVD is difficult to distinguish observationally. However, the fact that treatment of acute myocardial infarction with blood derived progenitor cells by intracoronary infusion increased myocardial viability in the infarct zone^62^ supports the hypothesis that disturbed ECFC function is not only indication of ED, but contributes to CVD by an impaired capacity to repair and maintain the endothelial layer.

We did not identify an effect of maternal weight gain or BMI, suggesting that maternal adipose tissue-derived paracrine factors do not affect neonatal ECFC outgrowth. Analyzing outgrowth of neonatal cord blood ECFC from 27 donors with maternal pre-pregnancy BMI ranging from 18 to 30 kg/m^2^, Moreno-Luna et al. observed a positive correlation between maternal BMI and the number of ECFC colonies, without differences in cell phenotype and function.^63^ The absence of such correlation in our study despite of a similar BMI range, may underlie differences in the isolation protocol: Freezing of MNC prior to ECFC isolation as performed by Moreno-Luna et al. may represent an additional selection step that promotes the growth of cells with a higher survival capacity. Thus, as a hypothesis, ECFC from donors with high BMI may be more robust and have a higher survival rate. In adults, however, BMI is negatively correlated with the number of circulating EPC,^35,64^ which also show reduced proliferation following initial outgrowth.^64^

We identified delivery mode as an important confounder. In fact, soluble endothelial markers are more frequent in cord blood after spontaneous vaginal delivery compared to elective C-section.^65^ Also Baker et al. reported reduced cord blood ECFC numbers after birth by C-section vs vaginal birth, with no difference between planned and emergent C-section in a study cohort of 62 preterm babies with and without pregnancy pathologies and further medical complications.^66^ The authors explained this difference by perinatal stress induced by vaginal birth leading to an increased release of angiogenic progenitor cells. The fact that the same finding is present in our full-term cohort highlights the physiological role of ECFC release at delivery. We did not detect a correlation between gestational age and ECFC number (not shown), which was identified in preterm infants^67^ and in a cohort with a gestational age range from 27 weeks to full term,^68^ respectively. The absent correlation in our study may be due to the fact that our cohort included only full-term pregnancies with deliveries between gestational weeks 37.4 and 41.7.

We see it as a limitation of our study that we do not have data on fetal insulin resistance, estrogen, IGF-1, and leptin. All these parameters are known to differ between male and female neonates^44,69^ and affect endothelial function.^50,51,70–72^ Thus, they may contribute to the observed sexual dimorphism in ECFC outgrowth.

This study highlights that not only do severe metabolic derangements affect fetal development, but parameters within the normal range also play a role. Whilst a wide array of studies investigated the effect of maternal hyperglycemia on the offspring’s short and long-term health, only very few studies investigated continuous glucose levels within the normal range. Yuan et al. demonstrated an impact of maternal FPG in pregnancy on endothelial function and cardiovascular risk markers in the offspring at age 6 years.^73^ Interestingly, this association existed only in the male offspring, which highlights the importance of sex-specific analysis in programming events.

The role of pre-and perinatal ECFC is not fully understood, however, a function in postnatal vasculogenesis and angiogenesis has been suggested.^74^ Additionally, the fast and active homing of hematopoietic progenitor cells from the neonatal circulation after birth^75^ suggests a significant role of ECFC in the neonatal vasculature, such as promotion of the rapid vascular growth, or support of major vascular remodeling events happening immediately after birth. A relation between number and function of neonatal ECFC and further development has not yet been investigated and we did not find any studies investigating the neonatal vascular system in regards to sex or maternal glycemia in pregnancy. However, a link between neonatal ECFC dysfunction in preterm delivery and adult risk for CVD has been hypothesized.^76^ In the light of the DOHaD (Developmental Origins of Health and Disease) paradigm, which describes the role of intrauterine influences in the development of diseases in later life,^77^ such long-term effects of adaptive and mal-programming events may be suggested and should be investigated in future studies.

## Supplementary information

Supplementary Figures

Supplementary Tables
