# Supplementary material for: Fetal sex and maternal fasting glucose affect neonatal cord blood-derived endothelial progenitor cells
Source: Pediatr Res. 2022 Feb 18;92(6):1590–7. doi: 10.1038/s41390-022-01966-4 (PMC9771817; doi:10.1038/s41390-022-01966-4)
Supplement: Supplementary file 1 — Supplementary Figures [file 41390_2022_1966_MOESM1_ESM.pdf]

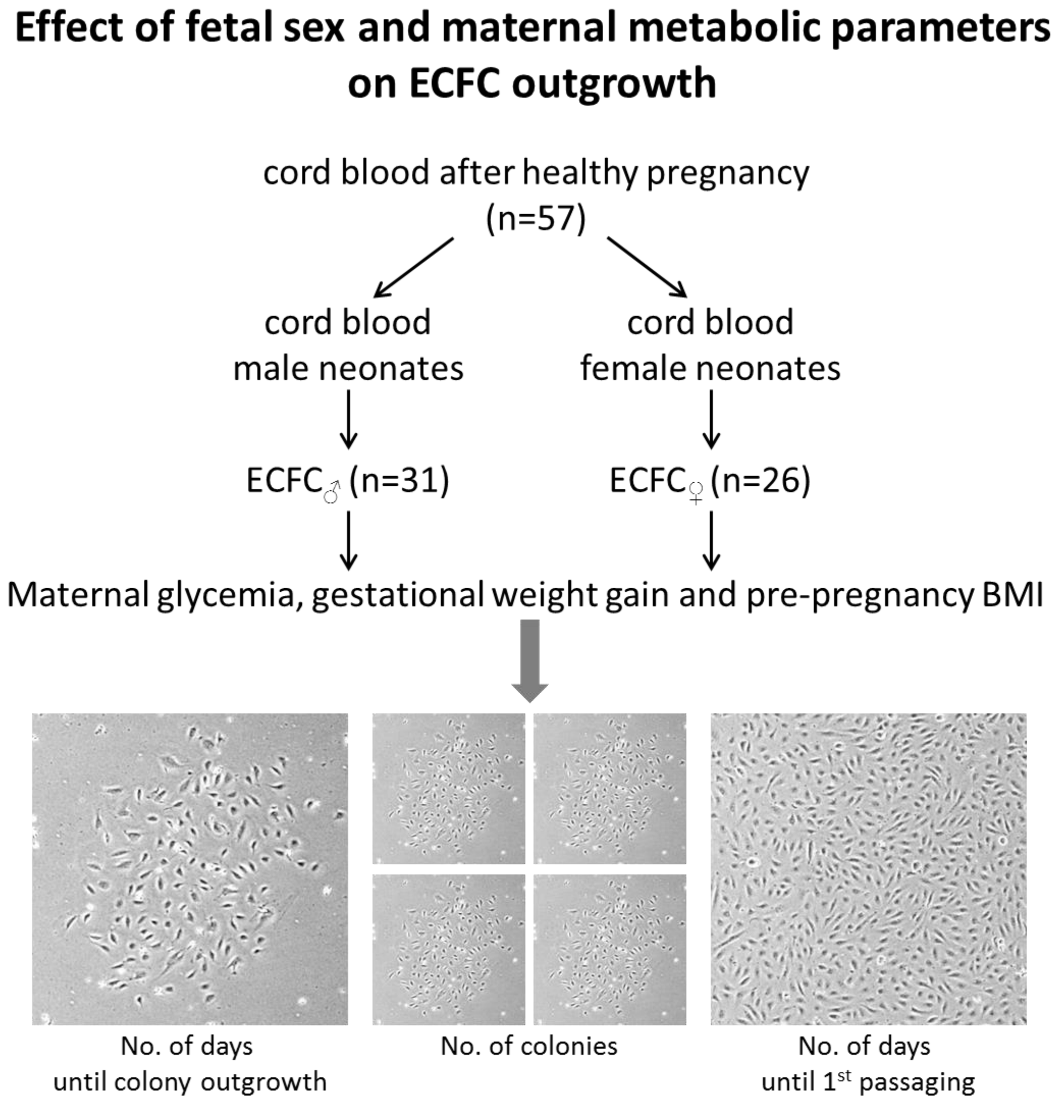

Supplementary Figure 1. Experimental study design.

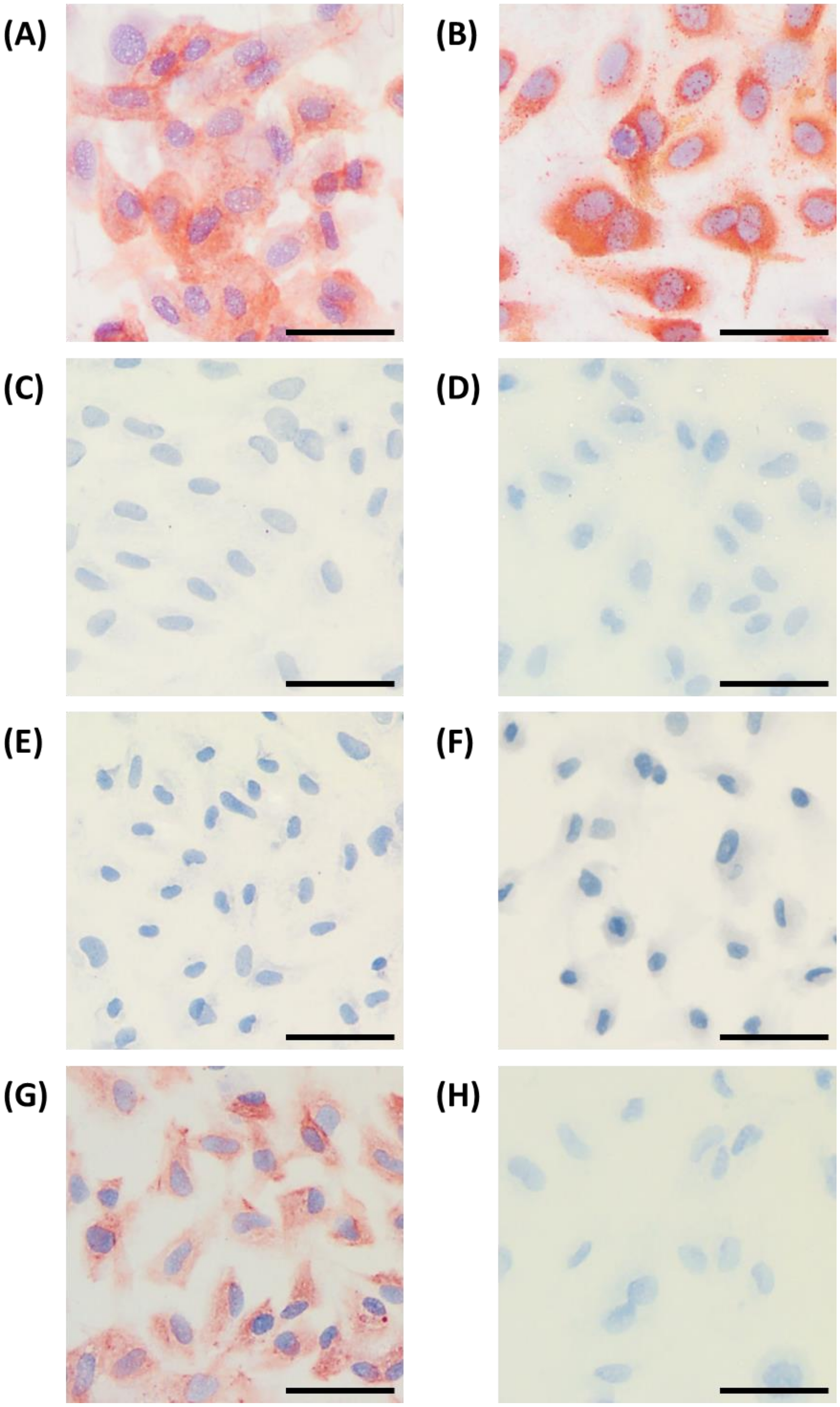

Supplementary Figure 2. Phenotype of cord blood ECFC as determined by immunocytochemistry. Cells were analyzed for surface expression of endothelial cell markers (CD31 (**A**), VWF (**B**)), muscle cell markers (SMA (**C**), Desmin (**D**)) and fibroblast markers (CD90 (**E**), TE-7 (**F**)). Vimentin (**G**), a protein present in all cells, served as positive control, mouse IgG1 (**H**) as negative control. Depicted images represent a typical expression pattern and show specific antibody staining (red) vs staining of the nuclei (blue). Scale bar = 50 µm.

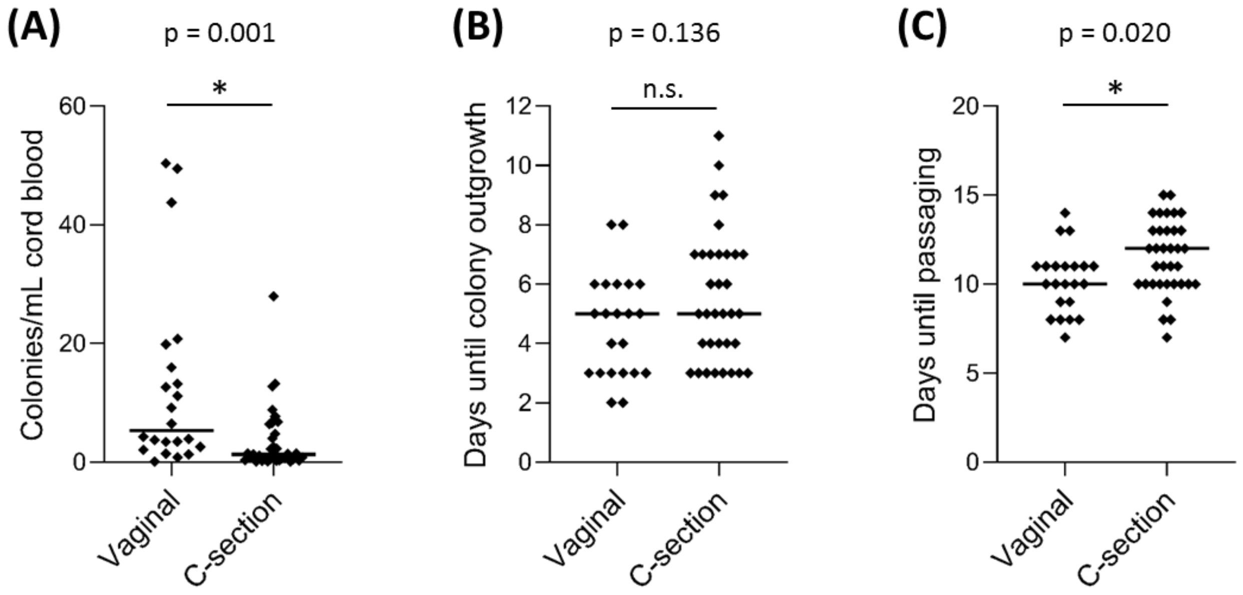

Supplementary Figure 3. Confounding factors for ECFC outgrowth. (**A**) Number of colonies in ECFC after vaginal delivery and C-section. (**B**) Days until colony outgrowth in ECFC after vaginal delivery and C-section. (**C**) Days until reaching confluency and passaging in ECFC after vaginal delivery and C-section. Data were analyzed via Mann-Whitney *U* test. n(vaginal delivery)=22, n(C-section)=35

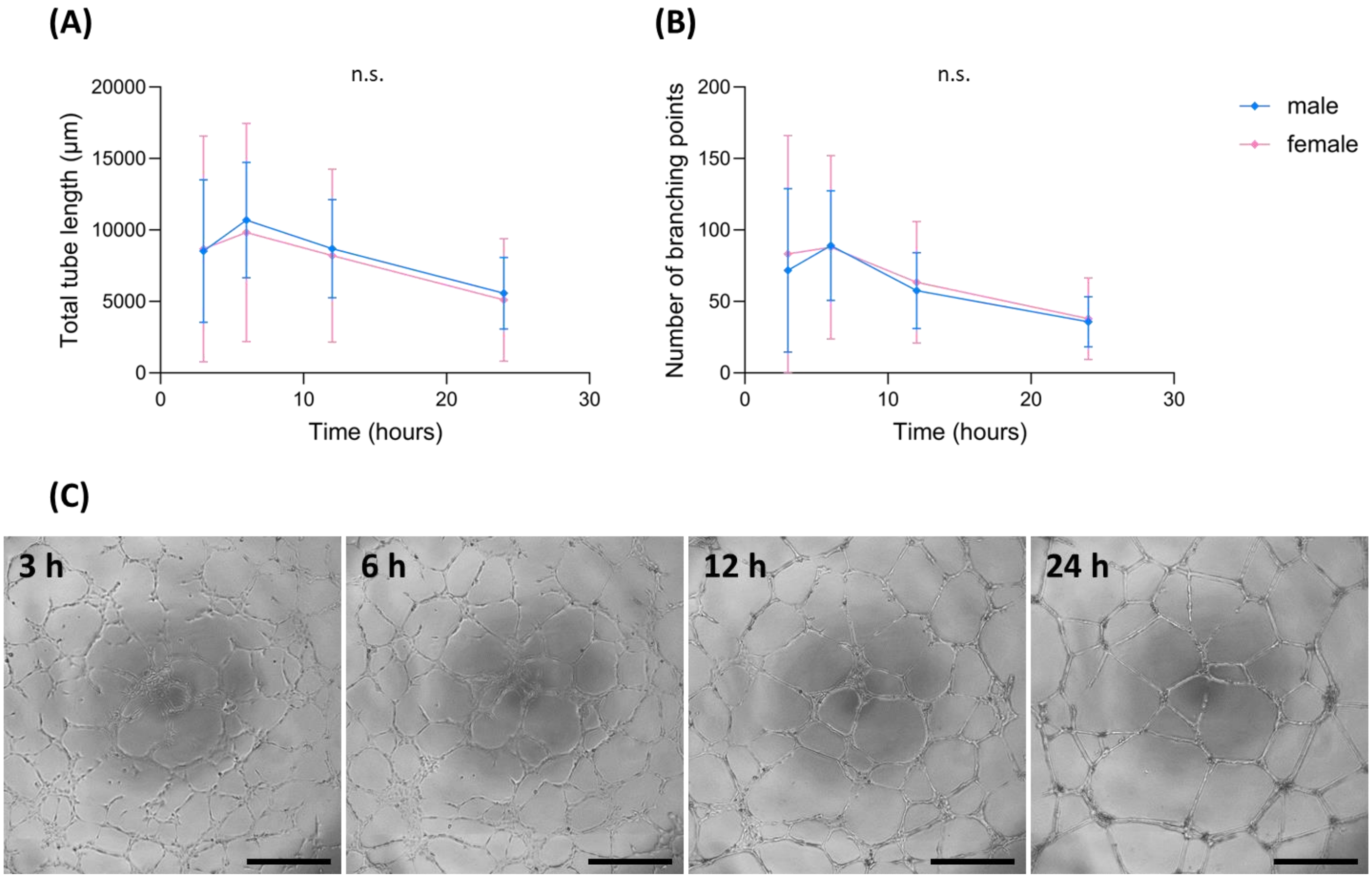

Supplementary Figure 4. Network formation assay on Matrigel. (**A**) Total tube length in male vs female ECFC. (**B**) Number of branching points in male vs female ECFC. (**C**) Depicted images represent a typical network formation over time. Data were analyzed via Student's t-test. n(male)=12, n(female)=11; Scale bar = 500 µm.
