# Supplementary material for: Fetal sex and maternal fasting glucose affect neonatal cord blood-derived endothelial progenitor cells
Source: Pediatr Res. 2022 Feb 18;92(6):1590–7. doi: 10.1038/s41390-022-01966-4 (PMC9771817; doi:10.1038/s41390-022-01966-4)
Supplement: Supplementary file 2 — Supplementary Tables [file 41390_2022_1966_MOESM2_ESM.pdf]

Supplementary Table 1. Antibodies used for flow cytometry. All antibodies originate from mouse species.

**Marker**	**Label**	**Manufacturer/Ord. No.**
**CD14**	FITC	Miltenyi Biotec/130-080-701
**CD31**	FITC	BD Pharmingen/560984
**CD34**	PE-Cy7	Beckman Coulter/A21691
**CD45**	PE	BD Pharmingen/555483
**CD90**	APC	BD Pharmingen/561971
**CD133**	APC	Miltenyi Biotec/130-098-829
**CD144**	PerCP-Cy5.5	BD Pharmingen/561566
**CD146**	PE	BD Pharmingen/561013
**CD309**	PE	BD Pharmingen/560494
**Tie-2**	APC	R&D/FAB3131A
**Isotype Controls**		
	APC	BD Pharmingen/555751
	FITC	BD Pharmingen/555748
	PE	BD Pharmingen/556027
	PE-Cy7	Beckman Coulter/737662
	PerCP-Cy5.5	BD Pharmingen/550795

Supplementary Table 2. Antibodies used for immunocytochemistry.

Marker	Manufacturer/Ord.	Isotype
**CD31**	Monosan/MON6002-1	Mouse IgG1
**VWF**	Dako/A0082	Polyclonal
**CD90**	Dianova/DIA100	Mouse IgG1
**TE-7**	Millipore/CBL271	Mouse IgG1
**SMA**	Dako/M0851	Mouse IgG2a
**Desmin**	Dako/M0760	Mouse IgG1
**Vimentin**	Dako/M0725	Mouse IgG1
**Negative**	Dako/X0931	Mouse IgG1

Supplementary Table 3. Network formation assay on Matrigel.

		Branching points (number)				Total tube length (µm)			
		3h	6h	12h	24h	3h	6h	12h	24h
Male		71.8±57.2	89.0±38.5	57.6±26.6	35.8±17.6	8531±4989	10697±4033	8696±3446	5583±2511
Female		83.2±82.9	87.9±64.3	63.4±42.5	37.9±28.5	8668±7911	9826±7631	8215±6057	5115±4283
	p	0.701	0.959	0.699	0.830	0.960	0.740	0.820	0.756
FGP	r	0.059	0.112	0.066	0.087	0.056	0.038	-0.007	-0.018
	p	0.789	0.610	0.765	0.692	0.799	0.864	0.974	0.934

Data are presented as mean ± SD. Statistical differences are calculated by unpaired Student's t-test (male vs female) or by Pearson correlation (FPG). FPG: Fasting plasma glucose.

Supplementary Table 4. Correlation of outgrowth parameters with maternal metabolic parameters in the entire cohort and in the groups with male and female neonates separately.

	entire cohort		male		female	
	OR (CI)	p	OR (CI)	p	OR (CI)	p
Fasting plasma glucose (oGTT 0 h)						
Colonies/mL	0.97 (0.91; 1.03)	0.353	0.98 (0.90; 1.06)	0.604	0.97 (0.87; 1.07)	0.497
Days until outgrowth	**1.02 (1.00; 1.04)**	**0.030**	**1.03 (1.00; 1.05)**	**0.029**	1.01 (0.98; 1.04)	0.405
Days until passaging	1.00 (1.00;1.01)	0.189	1.01 (1.00; 1.02)	0.223	1.00 (0.99; 1.02)	0.599
Post-load glycemia (oGTT 1 h)						
Colonies/mL	0.99 (0.98; 1.00)	0.201	0.99 (0.97; 1.01)	0.518	1.00 (0.97; 1.02)	0.659
Days until outgrowth	1.00 (1.00; 1.01)	0.291	1.00 (1.00; 1.01)	0.727	1.00 (1.00; 1.01)	0.759
Days until passaging	1.00 (1.00; 1.00)	0.245	1.00 (1.00; 1.00)	0.849	1.00 (1.00; 1.00)	0.495
Post-load glycemia (oGTT 2 h)						
Colonies/mL	1.01 (0.99; 1.03)	0.200	1.02 (1.00; 1.04)	0.095	1.01 (0.98; 1.04)	0.502
Days until outgrowth	1.00 (0.99; 1.00)	0.241	1.00 (0.99; 1.00)	0.409	1.00 (0.99; 1.00)	0.164
Days until passaging	1.00 (1.00; 1.00)	0.114	1.00 (1.00; 1.00)	0.164	1.00 (0.99; 1.00)	0.131
Pre-pregnancy BMI						
Colonies/mL	1.01 (0.91; 1.11)	0.878	1.01 (0.90; 1.15)	0.806	1.02 (0.86; 1.20)	0.836
Days until outgrowth	1.00 (0.97; 1.02)	0.765	1.01 (0.97; 1.04)	0.701	0.97 (0.94; 1.01)	0.171
Days until passaging	1.00 (0.99; 1.01)	0.660	1.00 (0.98; 1.01)	0.572	1.00 (0.98; 1.02)	0.731
Gestational weight gain						
Colonies/mL	1.02 (0.95; 1.10)	0.494	1.03 (0.95; 1.12)	0.466	0.98 (0.84; 1.14)	0.755
Days until outgrowth	1.00 (0.98; 1.02)	0.734	0.99 (0.97; 1.02)	0.572	1.02 (0.98; 1.05)	0.396
Days until passaging	1.00 (0.99; 1.00)	0.324	1.00 (0.99; 1.01)	0.438	1.00 (0.98; 1.02)	0.707

Data were analyzed by linear regression analysis and are presented as unstandardized regression coefficient B, that requires interpretation as odds ratio (OR), and 95% confidence intervals (CI). Bolt letters indicate $p<0.05$. oGTT: oral glucose tolerance test.
